# Systematic review of the literature on vitamin A and memory

**DOI:** 10.1590/S1980-57642012DN06040005

**Published:** 2012

**Authors:** Yara Dadalti Fragoso, Niklas Söderberg Campos, Breno Faria Tenrreiro, Fernanda Jussio Guillen

**Affiliations:** 1 Head of the Department of Neurology, Universidade Metropolitana de Santos, SP, Brazil.; 2 Medical Student, Universidade Metropolitana de Santos, SP, Brazil.

**Keywords:** vitamin A, retinol, retinoic acid, memory, cognition, Alzheimer

## Abstract

**Background:**

Over the last 30 years, a variety of studies reporting the effects of vitamin A on memory have been published.

**Objective:**

To perform a rigorous systematic review of the literature on vitamin A and memory in order to organize evidence-based data on the subject.

**Methods:**

Four authors carried out the systematic review in accordance with strict guidelines. The terms "vitamin A" OR "retinol" OR "retinoic acid" AND "memory" OR "cognition" OR "Alzheimer" were searched in virtually all medical research databases.

**Results:**

From 236 studies containing the key words, 44 were selected for this review, numbering 10 reviews and 34 original articles. Most studies used animal models for studying vitamin A and cognition. Birds, mice and rats were more frequently employed whereas human studies accounted for only two reports on brain tissue from autopsies and one on the role of isotretinoin in cognition among individuals taking this medication to treat acne.

**Conclusion:**

Vitamin A may be an important and viable complement in the treatment and prevention of Alzheimer's disease. Clinical trials are imperative and, at present, there is no evidence-based data to recommend vitamin A supplementation for the prevention or treatment of Alzheimer's disease.

## Abstract (pt)

**Introdução:**

Uma variedade de artigos relatando efeitos da vitamina A na memória foram publicados nos últimos 30 anos.

**Objetivo:**

Realizar uma revisão sistemática rigorosa da literatura sobre vitamina A e memória, visando organizar os dados com base em evidência.

**Métodos:**

Quatro autores realizaram a revisão sistemática de acordo com recomendações específicas para tal. Os termos "vitamin A" OR "retinol" OR "retinoic acid" AND "memory" OR "cognition" OR "Alzheimer" foram utilizados na pesquisa de praticamente todas as bases de dados de publicações médicas.

**Resultados:**

Dos 236 artigos contendo as palavras específicas de busca, 44 foram selecionados para esta revisão, sendo 10 revisões e 34 artigos originais. A maioria dos estudos utilizou modelos animais para avaliar vitamina A e cognição. Pássaros, camundongos e ratos foram mais frequentemente utilizados, enquanto estudos com humanos foram apenas dois relatando dados de necrópsia de cérebro humano e um sobre o papel da isotretinoína na cognição de indivíduos que estavam usando esta medicação para tratar acne.

**Conclusão:**

Vitamina A pode ser um complemento importante e viável no tratamento e prevenção da doença de Alzheimer. Estudos clínicos são imperativos e, no momento, não existe evidência científica que recomende suplementação de vitamina A na prevenção ou tratamento da doença de Alzheimer.

## INTRODUCTION

Vitamin A is an essential component of the human diet. It is derived from vitamin-A-rich foods as well as from foods containing beta-carotene, composed of two retinol molecules.^1^ Retinoic acid (RA) is the active metabolite of vitamin A and is a critical signaling molecule for both the developing and adult central nervous system (CNS). RA is synthesized more by the CNS than by any other organ and has long been recognized as a crucial factor for controlling the differentiation program of certain cells.^2^ RA is produced from the irreversible oxidation promoted by retinaldehyde dehydrogenase (RALDH). This enzyme is present in three distinctive isoforms (RALDH1, RALDH2 and RALDH3), which display non-overlapping tissue-specific patterns of expression during embryogenesis.^3^ Vitamin A deficiency could result in impaired cellular differentiation, reduced resistance to infection, anemia and, ultimately, death. In fact, vitamin A deficiency is a serious health problem in developing nations.^4,5^

One of the most interesting aspects of RA in the brain is in relation to memory. The complexity of RA involvement in memory is such that either too much or too little can result in similar deficits in learning behaviors.^6^ Retinoic acid is broadly implicated in neurogenesis, plasticity, cell differentiation and synaptic connectivity, but RA levels must be maintained at moderate levels through complex feedback control for appropriate learning.^6^ It might be said that excessive plasticity could be detrimental for effective learning and consolidation of very specific patterns and tasks. Most knowledge on vitamin A and memory is derived from animal studies and this field of research is set to remain open for many years to come. However, even without evidence-based data, the appeal of vitamin supplementation for prevention and treatment of cognitive dysfunction in adults can be problematic. In children, vitamin A supplementation has saved many lives^7,8^ yet has endangered others.^9,10^ In adults supplementing their diets with vitamin capsules, the problem has yet to be properly assessed. Whether vitamin A is related to memory functions and whether its supplementation can yield benefits in a clinical setting remains to be established. A case-control study in the early 1990s suggested that Alzheimer's disease might be associated to low levels of vitamin A and beta-carotene.^11^ Twenty years on, and with much more research having been carried out in this field,^12^ there is still a lack of clinical trials assessing the effects of vitamin A and cognition.

The present systematic review was conducted with the aim of collecting and organizing data in the literature on the subject of vitamin A and memory.

## METHODS

This systematic review of the literature followed the strict guidelines set forth by the PRISMA group.^13^ There were no meta-analyses of the data since the present report intended to be essentially descriptive and qualitative. No approval from the Ethics Committee was required since the study was carried out solely with published data from the world literature. The present study was registered as a scientific project at the Research Center of Universidade Metropolitana de Santos, SP, Brazil.

Using the PICO framework,^14^ the authors independently searched for the terms "vitamin A" OR "retinol" OR "retinoic acid" AND "memory" OR "cognition" OR "Alzheimer" in the following databases: Medline, Pubmed, Scopus, Index Medicus, Biomed Central, Ebsco Fulltext, LILACS, Scielo and the Cochrane Database of Systematic Reviews. Abstracts of articles in any language that contained these words in English (in the title, key words or abstract) were independently reviewed by the authors. The latest date of publication for inclusion of articles in the study was 10^th^ July 2012.

The inclusion criterion was to evaluate papers presenting original work and reviews on aspects of memory that were related to vitamin A. Studies on animals and humans were both included. However, research concentrating only on cell line cultures was not included in this review.

Studies reporting indirect evidence of the role of vitamin A and memory, for example the effect of this vitamin on the formation of plaques or fibrils, were also considered to be relevant to the present review, and were therefore included.

Abstracts from scientific meetings, anecdotal case reports, duplicate papers and editorials were excluded. Papers reporting exclusively on social behavior and/or sleep patterns relating to vitamin A were also excluded. Studies reporting on the role of RA in regenerating axolotl limbs or immunological lymphocyte memory patterns were also excluded, since these were not related to the "memory" aspect of the present paper (which was essentially one of cognition).

A recent systematic review has concluded that isotretinoin (13-cis-RA) is related to severe mood disorders in humans.^15^ Therefore, all papers reporting on the psychiatric aspects of vitamin A were excluded from the present review.

Papers reporting on the effect of "carotenoids" and/or "antioxidants" in general were also not included, as the present review concentrated exclusively on vitamin A.

All abstracts obtained retrieved using the specific key words were read individually by three of the authors. Once they had agreed on the abstracts that complied with the inclusion criteria, the full papers were obtained. Reference lists from the selected papers were checked to search for other possible relevant publications. The articles selected for the systematic review were read and summarized by the author who had not participated in the initial selection of abstracts.

## RESULTS

The initial search yielded 236 papers containing the specific key words. From these studies, 88 were selected for full reading of the text. The remaining 148 papers did not fulfill the inclusion criteria. Of the 88 papers initially selected, 44 were selected for this review.

The period of the literature search was open, but the initial study included in the present review dated from 1997, when Enderlin et al^15^ reported that RA could be involved in the alterations of synaptic plasticity observed in elderly mice. At the same time, Connor & Sidell^16^ reported on the activity of RALDHs in the human hippocampus in controls and individuals with Alzheimer's disease. In 1998, Chiang et al.^18^ reported a pattern of RA and plasticity by studying spatial learning and memory tasks. These authors stated that a novel and unexpected role for vitamin A was thus observed for higher cognitive functions. These studies published in the late 1990s were the first included in the present review. After these studies, many others ensued and a summary of these is given in Table 1. Ten reviews were identified among the publications,^12,29,30,32,34,37,45-47,51^ while 34 papers presented original data.^15-28,31,33-36,38-44,48-50,52-57^

The animal models for studying vitamin A and cognition were birds, mice and rats. The human studies consisted of two reports on brain tissue from autopsies^16,54^ and one paper on the role of isotretinoin in cognition among individuals taking this medication to treat acne.^50^

## DISCUSSION

The studies on vitamin A and cognition, according to this systematic review, point to a role for RA in higher mental function. From learning simple tasks to establishing memory patterns, animals performed better with supplementation of vitamin A and worse in its absence from the diet. However, excessive amounts of vitamin A had a detrimental effect on the pattern of vocally learned processes in birds. This is an important point to consider, since some "treatments" for cognitive dysfunction in humans have historically envisaged excessive doses of vitamins.

From the first studies^17^ to the latest papers,^57^ long-term potentiation (LTP) and long-term depression (LTD) in the hippocampus appear to be positively affected by RA. LTP and LTD are cellular mechanisms for learning and memory and are therefore critical for cognition. It has been demonstrated that RA can increment LTP and LTD,^56^ inhibit the deposition of beta amyloid,^25,26,38^ while also have anti-oxidative, cell protective and anti-aggregation effects.^51^ At least in mice, RA seems to be distributed differently in the layers of the dentate gyrus of the hippocampus,^57^ and the functional implications of this finding could be linked to RA-regulated transcription.

RA receptors and related enzymes are present in the human hippocampus^16,54^ and may be significantly affected by Alzheimer's disease.^16^ Therapeutic effects of vitamin A supplementation for protection against this dementia are a real possibility and clinical trials should be carried out in order to assess the efficacy and safety of vitamin A supplementation for the prevention and treatment of Alzheimer's disease. This matter must be investigated with scientific rigor, as the indiscriminate use of vitamin A supplementation for treatment (or prevention) of Alzheimer's disease is currently not based on scientific and/or medical evidence.

To conclude, vitamin A has been shown to have positive effects on cognition. Data on humans are scarce and no controlled studies have yet been carried out. Therefore, no recommendation for dietary supplementation with vitamin A can be proposed at this time with the purpose of preventing or treating Alzheimer's disease and/or other dementias. However, results from the studies summarized in this systematic review are encouraging and suggest a potential therapeutic effect of this vitamin in humans.

## References

[1] Bendich A, Olson JA (1989). Biological actions of carotenoids. FASEB J.

[2] Napoli JL (1999). Interactions of retinoid binding proteins and enzymes in retinoid
metabolism. Biochim Biophys Acta.

[3] Mic FA, Haselbeck RJ, Cuenca AE, Duester G (2002). Novel retinoic acid generating activities in the neural tube and
heart identified by conditional rescue of Raldh2 null mutant
mice. Development.

[4] Arlappa N (2011). Vitamin A deficiency is still a public health problem in
India. Indian Pediatr.

[5] Jiang J, Toschke AM, von Kries R, Koletzko B, Lin L (2006). Vitamin A status among children in China. Public Health Nutr.

[6] Olson CR, Mello CV (2010). Significance of vitamin A to brain function, behavior and
learning. Mol Nutr Food Res.

[7] Imdad A, Yakoob MY, Sudfeld C, Haider BA, Black RE, Bhutta ZA (2011). Impact of vitamin A supplementation on infant and childhood
mortality. BMC Public Health.

[8] Barbosa Chagas C, Ramalho A, de Carvalho Padilha P, Delia Libera B, Saunders C (2011). Reduction of vitamin A deficiency and anemia in pregnancy after
implementing proposed prenatal nutritional assistance. Nutr Hosp.

[9] Lam HS, Chow CM, Poon WT (2006). Risk of vitamin A toxicity from candy-like chewable vitamin
supplements for children. Pediatrics.

[10] Duerbeck NB, Dowling DD (2012). Vitamin A: too much of a good thing?. Obstet Gynecol Surv.

[11] Zaman Z, Roche S, Fielden P, Frost PG, Niriella DC, CAyley AC (1992). Plasma concentrations of vitamins A and E and carotenoids in
Alzheimer's disease. Age ageing.

[12] Obulesu M, Dowlathabad MR, Bramhachari PV (2011). Carotenoids and Alzheimer's disease: an insight into therapeutic
role of retinoids in animal models. Neurochem Int.

[13] Moher D, Liberati A, Tetzlaff J, Altman DG, The PRISMA Group (2009). Preferred Reporting Items for Systematic Reviews and
Meta-Analyses: The PRISMA Statement. Open Med.

[14] Schardt C, Adams MB, Owens T, Keitz S, Fontelo P (2007). Utilization of the PICO framework to improve searching PubMed for
clinical questions. BMC Med Inform Decis Mak.

[15] Enderlin V, Pallet V, Alfos S, Dargelos E, Jaffard R, Garcin H, Higueret P (1997). Age-related decreases in mRNA for brain nuclear receptors and
target genes are reversed by retinoic acid treatment. Neurosci Lett.

[16] Connor MJ, Sidell N (1997). Retinoic acid synthesis in normal and Alzheimer diseased brain
and human neural cells. Mol Chem Neuropathol.

[17] Chiang MY, Misner D, Kempermann G (1998). An essential role for retinoid receptors RARbeta and RXRgamma in
long-term potentiation and depression. Neuron.

[18] Denisenko-Nehrbass NI, Jarvis E, Scharff C, Nottebohm F, Mello CV (2000). Site-specific retinoic acid production in the brain of adult
songbirds. Neuron.

[19] Alfos S, Boucheron C, Pallet V (2001). A retinoic acid receptor antagonist suppresses brain retinoic
acid receptor overexpression and reverses a working memory deficit induced
by chronic ethanol consumption in mice. Alcohol Clin Exp Res.

[20] Etchamendy N, Enderlin V, Marighetto A (2001). Alleviation of a selective age-related relational memory deficit
in mice by pharmacologically induced normalization of brain retinoid
signaling. J Neurosci.

[21] Misner DL, Jacobs S, Shimizu Y (2001). Vitamin A deprivation results in reversible loss of hippocampal
long-term synaptic plasticity. Proc Natl Acad Sci USA.

[22] Cocco S, Diaz G, Stancampiano R, Diana A (2002). Vitamin A deficiency produces spatial learning and memory
impairment in rats. Neuroscience.

[23] Etchamendy N, Enderlin V, Marighetto A, Pallet V, Higueret P, Jaffard R (2003). Vitamin A deficiency and relational memory deficit in adult mice:
relationships with changes in brain retinoid signalling. Behav Brain Res.

[24] Goodman AB, Pardee AB (2003). Evidence for defective retinoid transport and function in late
onset Alzheimer's disease. Proc Natl Acad Sci USA.

[25] Corcoran JP, So PL, Maden M (2004). Disruption of the retinoid signalling pathway causes a deposition
of amyloid beta in the adult rat brain. Eur J Neurosci.

[26] Ono K, Yoshiike Y, Takashima A, Hasegawa K, Naiki H, Yamada M (2004). Vitamin A exhibits potent antiamyloidogenic and
fibril-destabilizing effects in vitro. Exp Neurol.

[27] Craft NE, Haitema TB, Garnett KM, Fitch KA, Dorey CK (2004). Carotenoid, tocopherol, and retinol concentrations in elderly
human brain. J Nutr Health Aging.

[28] Wietrzych M, Meziane H, Sutter A (2005). Working memory deficits in retinoid X receptor gamma-deficient
mice. Learn Mem.

[29] Lane MA, Bailey SJ (2005). Role of retinoid signalling in the adult brain. Prog Neurobiol.

[30] Goodman AB (2006). Retinoid receptors, transporters, and metabolizers as therapeutic
targets in late onset Alzheimer disease. J Cell Physiol.

[31] Hernández-Pinto AM, Puebla-Jiménez L, Arilla-Ferreiro E (2006). A vitamin Afree diet results in impairment of the rat hippocampal
somatostatinergic system. Neuroscience.

[32] McCaffery P, Zhang J, Crandall JE (2006). Retinoic acid signaling and function in the adult
hippocampus. J Neurobiol.

[33] Mao CT, Li TY, Qu P, Zhao Y, Wang R, Liu YX (2006). Effects of early intervention on learning and memory in young
rats of marginal vitamin A deficiency and its mechanism. Zhonghua Er Ke Za Zhi.

[34] Dräger UC (2006). Retinoic acid signaling in the functioning brain. Sci STKE.

[35] Kheirvari S, Uezu K, Sakai T (2006). Increased nerve growth factor by zinc supplementation with
concurrent vitamin A deficiency does not improve memory performance in
mice. J Nutr Sci Vitaminol (Tokyo).

[36] Stancampiano R, Carta M, Fadda F (2007). Vitamin A deficiency affects neither frontocortical acetylcholine
nor working memory. Neuroreport.

[37] Tafti M, Ghyselinck NB (2007). Functional implication of the vitamin A signaling pathway in the
brain. Arch Neurol.

[38] Ono K, Yamada M (2007). Vitamin A potently destabilizes preformed alphasynuclein fibrils
in vitro: implications for Lewy body diseases. Neurobiol Dis.

[39] Ding Y, Qiao A, Wang Z (2008). Retinoic acid attenuates beta-amyloid deposition and rescues
memory deficits in an Alzheimer's disease transgenic mouse
model. J Neurosci.

[40] Bonnet E, Touyarot K, Alfos S, Pallet V, Higueret P, Abrous DN (2008). Retinoic acid restores adult hippocampal neurogenesis and
reverses spatial memory deficit in vitamin A deprived rats. PLoS One.

[41] Mingaud F, Mormede C, Etchamendy N (2008). Retinoid hyposignaling contributes to aging-related decline in
hippocampal function in shortterm/ working memory organization and long-term
declarative memory encoding in mice. J Neurosci.

[42] Fonzo LS, Golini RS, Delgado SM (2009). Temporal patterns of lipoperoxidation and antioxidant enzymes are
modified in the hippocampus of vitamin A-deficient rats. Hippocampus.

[43] Yang Z, Xi J, Li J, Qu W (2009). Biphasic effect of citral, a flavoring and scenting agent, on
spatial learning and memory in rats. Pharmacol Biochem Behav.

[44] Munetsuna E, Hojo Y, Hattori M (2009). Retinoic acid stimulates 17betaestradiol and testosterone
synthesis in rat hippocampal slice cultures. Endocrinology.

[45] Lee HP, Casadesus G, Zhu X (2009). All-trans retinoic acid as a novel therapeutic strategy for
Alzheimer's disease. Expert Rev Neurother.

[46] Luo T, Wagner E, Dräger UC (2009). Integrating retinoic acid signaling with brain
function. Dev Psychol.

[47] Olson CR, Mello CV (2010). Significance of vitamin A to brain function, behavior and
learning. Mol Nutr Food Res.

[48] Zhang X, Chen K, Chen J, Liu YX, Qu P, Li TY (2011). Effect of marginal vitamin A deficiency during pregnancy on
retinoic acid receptors and N-methyl-D-aspartate receptor expression in the
offspring of rats. J Nutr Biochem.

[49] Wietrzych-Schindler M, Szyszka-Niagolov M, Ohta K (2011). Retinoid x receptor gamma is implicated in docosahexaenoic acid
modulation of despair behaviors and working memory in mice. Biol Psychiatry.

[50] Ormerod AD, Thind CK, Rice SA, Reid IC, Williams JH, McCaffery PJ (2012). Influence of isotretinoin on hippocampal-based learning in human
subjects. Psychopharmacology (Berl).

[51] Ono K, Yamada M (2012). Vitamin A and Alzheimer's disease. Geriatr Gerontol Int.

[52] Jiang W, Yu Q, Gong M (2012). Vitamin A deficiency impairs postnatal cognitive function via
inhibition of neuronal calcium excitability in hippocampus. J Neurochem.

[53] Golini RS, Delgado SM, Navigatore Fonzo LS, Ponce IT, Lacoste MG, Anzulovich AC (2012). Daily patterns of clock and cognition-related factors are
modified in the hippocampus of vitamin A-deficient rats. Hippocampus.

[54] Fragoso YD, Shearer KD, Sementilli A, de Carvalho LV, McCaffery PJ (2012). High expression of retinoic acid receptors and synthetic enzymes
in the human hippocampus. Brain Struct Funct.

[55] Guo M, Bryant J, Sultana S, Jones O, Royal W (2012). Effects of Vitamin A Deficiency and Opioids on Parvalbumin+
Interneurons in the Hippocampus of the HIV-1 Transgenic Rat. Curr HIV Res.

[56] Nomoto M, Takeda Y, Uchida S (2012). Dysfunction of the RAR/RXR signaling pathway in the forebrain
impairs hippocampal memory and synaptic plasticity. Mol Brain.

[57] Goodman T, Crandall JE, Nanescu SE (2012). Patterning of retinoic acid signaling and cell proliferation in
the hippocampus. Hippocampus.

